# Four New Sulfated Polar Steroids from the Far Eastern Starfish *Leptasterias ochotensis:* Structures and Activities

**DOI:** 10.3390/md13074418

**Published:** 2015-07-16

**Authors:** Timofey V. Malyarenko, Olesya S. Malyarenko (Vishchuk), Natalia V. Ivanchina, Anatoly I. Kalinovsky, Roman S. Popov, Alla A. Kicha

**Affiliations:** G.B. Elyakov Pacific Institute of Bioorganic Chemistry, Far East Branch of the Russian Academy of Sciences, Pr. 100-let Vladivostoku 159, 690022 Vladivostok, Russia; E-Mails: malyarenko-tv@mail.ru (T.V.M.); vishchuk87@gmail.com (O.S.M.); ivanchina@piboc.dvo.ru (N.V.I.); kaaniv@piboc.dvo.ru (A.I.K.); prs_90@mail.ru (R.S.P.)

**Keywords:** steroids, sulfated steroids, glycosides, starfish, *Leptasterias ochotensis*, cytotoxicity, neoplastic cell transformation, MAPK

## Abstract

Three new sulfated steroid monoglycosides, leptaochotensosides A–C (**1**–**3**), and a new sulfated polyhydroxylated steroid (**4**) were isolated from the alcoholic extract of the Far Eastern starfish *Leptasterias ochotensis*. The structures of compounds **1**–**4** were established by extensive nuclear magnetic resonance (NMR) and electrospray ionization mass spectrometry (ESIMS) analyses and chemical transformations. Although the isolated compounds did not show any apparent cytotoxicity against melanoma RPMI-7951 and breast cancer T-47D cell lines, leptaochotensoside A (**1**) demonstrated inhibition of T-47D cell colony formation in a soft agar clonogenic assay at nontoxic doses. In addition, this compound decreased the epidermal growth factor (EGF)-induced colony formation of mouse epidermal JB6 Cl41 cells. The cancer preventive action of **1** is realized through regulation of mitogen-activated protein kinase (MAPK) signaling pathway.

## 1. Introduction

Starfish (*Echinodermata*, *Asteroidea*) are characterized by a diversity of polar steroids, including polyhydroxylated steroids, structurally related mono-, bi- and triosides, and steroid oligoglycosides (asterosaponins) with carbohydrate chains comprising five or six sugars [1,2,3,4]. Free polyhydroxysteroids and their glycosides are highly oxygenated steroid compounds, having three to nine hydroxyl groups, and often occur in sulfated forms. Starfish polar steroids have been reported to show a wide spectrum of biological activities, including hemolytic, cytotoxic, antiviral, antibacterial, antifouling, neuritogenic, and antifungal effects [1,2,3,4]. Recently, some data on the cancer preventive and anticancer activities of starfish polar steroids were obtained. Several asterosaponins and steroid mono- or biosides were found to have anticancer properties showing strong *in vitro* cytotoxicity against different tumor cells [5]. For example, effective cytostatics, asterosaponin 1 and novaeguinoside II from the starfish *Culcita novaeguineae* induced apoptosis of human glioblastoma U87MG cells through several signaling transduction pathways [6,7]. Moreover, asterosaponin 1 inhibited the proliferation of A549 human lung cancer cells through induction of endoplasmic reticulum stress-associated apoptosis [8]. Polyhydroxysteroid glycosides isolated from the starfish *Anthenea chinensis* exhibited significant activity against promotion of tubulin polymerization *in vitro* inhibiting the proliferation of glioblastoma cells [9]. Leviusculoside G, steroid biglycoside from the starfish *Henricia leviuscila*, demonstrated anticarcinogenic action by the induction of p53-dependent apoptosis and inhibition of activator protein 1 (AP-1), nuclear factor kappa-light-chain-enhancer of activated B cells (NF-κB), and extracellular-signal-regulated protein kinases (ERKs) activities in human leukemia HL-60, THP-1, and mouse epidermal JB6 Cl41 cells [10]. Archasteroside B from the starfish *Archaster typicus* induced basal AP-1- and p53-, but not NF-κB-transcriptional activations in JB6 Cl41 cells [11]. Some asterosaponins and other steroid glycosides from the starfish *Hippasteria kurilensis*, *Asteropsis carinifera* and *Lethasterias fusca* exhibited a significant suppression of the human tumor HT-29, HCT-116, RPMI-7951, and T-47D cell colony formation in a soft agar clonogenic assay [12,13,14]. All these results indicate that further studies of the anticancer properties of polar steroids from starfish are necessary to be conducted.

Recently, we have established structures of six new asterosaponins, leptasteriosides A–F along with one new and one previously known asterogenins from the starfish *Leptasterias ochotensis* (order Forcipulatida, family Asteriidae) collected near Shantar Islands in the Sea of Okhotsk. Leptasteriosides A–C demonstrated a substantial suppression of colony formation of human melanoma RPMI-7951 and breast cancer T-47D cells [15].

Herein, we report the results of the structural elucidation of four new sulfated steroid compounds (**1**–**4**) from the fraction of sulfated polyxydroxysteroids and related glycosides from *L. ochotensis*. Moreover, we discuss the capabilities of **1**–**4** to inhibit colony formation of cancer RPMI-7951 and T-47D cells *ex vivo*, the suppressing influence of **1** on the epidermal growth factor (EGF)-induced colony formation of mouse epidermal JB6 Cl41 cells, and the molecular mechanism of cancer preventive effect of **1** implemented through regulation of mitogen-activated protein kinase (MAPK) signaling pathway.

## 2. Results and Discussion

**Figure 1:**
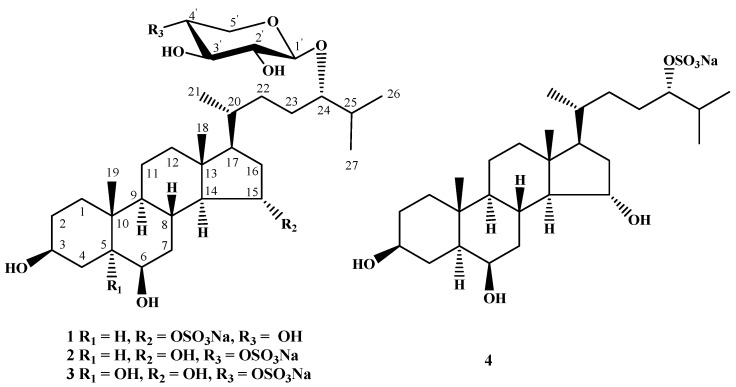
The structures of compounds **1**–**4** isolated from *L. ochotensis*.

The concentrated ethanol extract of *L. ochotensis* was subjected to sequential separation by chromatography on columns with Polyсhrom-1 and silica gel followed by high-performance liquid chromatography (HPLC) on semipreparative Diasfer-110-C18, Discovery C_18_ and analytical Diasfer-110-C18 columns to yield three new sulfated steroid monoglycosides, named as leptaochotensosides A–C (**1**–**3**), and one new sulfated tetrahydroxylated steroid **4** (Figure 1).

**Figure 2:**
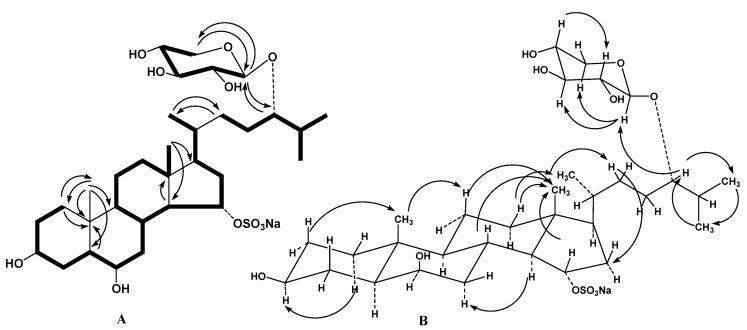
(**A**) ^1^H-^1^H Correlation spectroscopy (^1^H-^1^H COSY) and key heteronuclear multiple bond connectivity (HMBC) correlations for compound **1**; (**B**) Key nuclear Overhauser effect spectroscopy (NOESY) correlations for compound **1**.

The molecular formula of compound **1** was determined to be of С_32_Н_55_О_11_SNa from the [M + Na]^+^ sodiated adduct ion peak at *m*/*z* 693.3225 in the positive high resolution electrospray ionization mass spectrometry ((+)HRESIMS) and the [M − Na]^−^ ion peak at *m*/*z* 647.3485 in the negative high resolution electrospray ionization mass spectrometry ((−)HRESIMS). The fragment ion peaks at *m*/*z* 573 [(M + Na) − NaHSO_4_]^+^, 143 [Na_2_HSO_4_]^+^ in the (+)ESIMS/MS of the ion at *m*/*z* 693 [M + Na]^+^ and *m*/*z* 97 [HSO_4_]^−^ in the (−)ESIMS/MS of the ion at *m*/*z* 647 [M − Na]^−^ showed the presence a sulfate group in **1**. The ^1^H and ^13^C NMR spectra of the tetracyclic moiety of the aglycon of **1** showed the resonances of protons and carbons of two angular methyls CH_3_-18 and CH_3_-19 (δ_H_ 0.77 s, 1.04 s; δ_C_ 13.8, 16.3), two oxygenated methines CH-3 (δ_H_ 3.54 m; δ_C_ 72.4), CH-6 [δ_H_ 3.74 q (*J* = 2.2); δ_C_ 72.6], and one *O*-sulfated methine CH-15 [δ_H_ 4.49 td (*J* = 9.1, 3.2); δ_C_ 82.2], that were characteristic of a 3β,6β,15α-trihydroxysteroid nucleus sulfated at position C-15 [16]. The NMR spectra of aglycon side chain indicated the existence of three secondary methyls CH_3_-21 [δ_H_ 0.92 d (*J* = 6.7); δ_C_ 19.1], CH_3_-26 [δ_H_ 0.92 d (*J* = 6.7); δ_C_ 18.4], and CH_3_-27 [δ_H_ 0.92 d (*J* = 6.7); δ_C_ 18.5] and oxygenated CH-24 group (δ_H_ 3.34 m; δ_C_ 86.2) bearing an *O*-monosaccharide residue. These proton and carbon resonances were similar to those of a 24-*O*-glycosylated cholestane chain of fuscaside A earlier isolated from the starfish *Lethasterias fusca* [17]. The ^1^H NMR spectrum exhibited one resonance in the downfield region due to an anomeric proton of monosaccharide unit at δ_H_ 4.06 correlated in the HSQC experiment with a carbon signal at δ_C_ 105.0. The (+)ESIMS/MS of the ion [M + Na]^+^ at *m*/*z* 693 and the (−)ESIMS/MS of the ion [M − Na]^−^ at *m/z* 647 contained the fragment ion peaks corresponding to the loss of a pentose at *m*/*z* 543 [(M + Na) − С_5_H_10_O_5_]^+^ and *m*/*z* 515 [(M − Na) − С_5_H_8_O_4_]^−^, respectively. Thus, the NMR and mass spectral data indicated the presence of a pentose unit and a tetrahydroxylated cholestane aglycon in **1**. All the proton and carbon signals of **1** were assigned using 2D NMR experiments, including ^1^H-^1^H correlation spectroscopy (^1^H-^1^H COSY), heteronuclear single quantum connectivity (HSQC), heteronuclear multiple bond connectivity (HMBC), and nuclear Overhauser effect spectroscopy (NOESY) (Table 1 and Table 2, Supplementary Materials S1–S7). The ^1^H-^1^H COSY and HSQC correlations confirmed the corresponding sequences of protons at C-1 to C-8, C-8 to C-12 through C-9 and C-11, C-12 to C-18, C-8 to C-14, C-14 to C-17, C-17 to C-21, C-23 and to the end of the side chain (Figure 2A). The HMBC cross-peaks, such as H-1/C-10, C-19; H-5/C-10; H-14/C-13; H_3_-18/C-14, C-17; H_3_-19/С-1, С-5, С-9, С-10; H_3_-21/C-22; and H_2_-22/C-21 supported the overall structure of the steroid moiety of **1** (Figure 2A). The key NOESY cross-peaks confirmed the common 5α/10β/8β/9α/13β/14α steroid nucleus and the 3β,6β,15α-configurations of oxygenated substituents in **1** (Figure 2B).

**Table 1:** ^1^H (700.13 MHz) Nuclear magnetic resonance (NMR) chemical shifts of **1**–**4** in D_4_-methanol (CD_3_OD), at 30 °C, δ in ppm, *J* values in Hz.

Position	1	2	3	4
1	1.64 m; 0.97 m	1.71 m; 1.02 m	1.60 m; 1.32 m	1.73 m; 1.02 m
2	1.73 m; 1.42 m	1.74 m; 1.39 m	1.75 m; 1.48 m	1.74 m; 1.40 m
3	3.54 m	3.47 m	4.00 m	3.47 m
4	1.74 m 1.55 m	2.18 m 1.15 m	2.05 dd (13.0; 11.2) 1.54 m	2.18 brd (9.8) 1.16 m
5	1.12 m	1.00 m		1.00 m
6	3.74 q (2.2)	3.32 m	3.46 t (2.8)	3.31 m
7	2.27 td (14.5; 3.2) 1.26 m	2.28 brd (14.9) 1.05 m	1.85 m	2.28 td (12.7; 4.3) 1.06 m
8	2.00 m	1.64 m	1.93 m	1.65 m
9	0.72 m	0.70 m	1.40 m	0.70 m
10				
11	1.54 m; 1.40 m	1.53 m; 1.31 m	1.39 m; 1.32 m	1.54 m; 1.30 m
12	1.98 m; 1.23 m	1.95 brd (12.8) 1.22 m	1.95 brd (11.8) 1.23 m	1.95 m 1.22 m
13				
14	1.26 m	1.04 m	1.11 m	1.09 m
15	4.49 td (9.1; 3.2)	3.86 td (11.1; 5.7)	3.86 td (10.4; 3.3)	3.84 td (9.5; 3.4)
16	2.22 m; 1.92 m	1.89 m; 1.73 m	1.88 m; 1.72 m	1.90 m; 1.74 m
17	1.37 m	1.39 m	1.39 m	1.39 m
18	0.77 s	0.71 s	0.72 s	0.71 s
19	1.04 s	0.84 s	1.16 s	0.84 s
20	1.39 m	1.34 m	1.36 m	1.38 m
21	0.92 d (6.7)	0.93 d (7.0)	0.93 d (6.9)	0.94 d (7.0)
22	1.60 m; 1.04 m	1.58 m; 0.98 m	1.59 m; 0.99 m	1.58 m; 1.07 m
23	1.59 m; 1.36 m	1.56 m; 1.34 m	1.57 m; 1.33 m	1.70 m; 1.55 m
24	3.34 m	3.35 m	3.35 m	4.11 q (5.9)
25	1.84 m	1.87 m	1.86 m	2.00 m
26	0.92 d (6.7)	0.92 d (7.0)	0.92 d (6.6)	0.95 d (6.3)
27	0.92 d (6.7)	0.92 d (7.0)	0.92 d (6.6)	0.91 d (6.3)
1′	4.06 d (6.6)	4.25 d (7.7)	4.25 d (7.6)	
2′	3.21 dd (9.0; 7.3)	3.23 dd (9.2; 7.6)	3.23 dd (9.2; 7.6)	
3′	3.29 m	3.49 t (8.8)	3.48 t (9.0)	
4′	3.54 m	4.18 m	4.18 m	
5′	3.88 dd (11.5; 5.3) 3.14 dd (11.4; 10.0)	4.16 dd (10.5; 5.6) 3.28 m	4.16 dd (10.5; 5.5) 3.28 m	

m, multiplet; s, singlet; d, double; t, triplet; q, quartet; dd, doublet of doublets; td, triplet of doublets; brd, broad doublet.

The chemical shifts and coupling constants of H-1–H-5 of a pentose unit were determined by the irradiation of anomeric proton in the 1D total correlation spectroscopy (1D TOCSY) experiment. The coupling constant of the anomeric proton of monosaccharide unit (6.6 Hz) corresponded to the β-configuration of the glycosidic bond. The carbon and proton signals and corresponding coupling constants of monosaccharide unit coincided well with those of the terminal β-xylopyranosyl residue [17]. Acid hydrolysis of glycoside **1** with 2 M trifluoroacetic acid (TFA) followed by obtaining of chiral derivatives were carried out to ascertain an absolute stereochemistry of its monosaccharide unit. Alcoholysis of the monosaccharide by (*R*)-(−)-octanol followed by acetylation, gas chromatopgraphy (GC) analysis, and comparison with corresponding derivatives of standard monosaccharides allowed us to establish the d-configuration of the β-xylose. The attachment position of the β-d-xylopyranosyl residue to steroid aglycon was confirmed by the HMBC and NOESY spectra, where cross-peaks between H-1′ of Xyl_p_ and C-24, and, respectively, H-24 of the aglycon were observed. The chemical shift of H_3_-21 at δ_H_ 0.92 and the NOESY cross-peaks H_3_-18/H-20, H_3_-21/H_β_-12, and H-22/H_β_-16 were indicative of the (*R*)-configuration for the C-20 asymmetric center [18]. The (24*S*)-configuration was proposed by similarity of the ^13^C NMR data of the side chain of **1** with those of fuscaside A and other steroid 24-*O*-β-xylopyranosides isolated from starfish [17]. Based on the results, the structure of leptaochotensoside A (**1**) was determined as (24*S*)-24-*O*-(β-d-xylopyranosyl)-5α-cholestane-3β,6β,15α,24-tetraol 15-*O*-sulfate. Compound **1** contains the 15-*O*-sulfated 3β,6β,15α-trihydroxysteroid nucleus never earlier found in other steroid glycosides from starfish.

The molecular formula of compound **2** was determined to be of С_32_Н_55_О_11_SNa, the same as in the glycoside **1**. The fragment ion peaks at *m*/*z* 573 [(M + Na) − NaHSO_4_]^+^ in the (+)ESIMS/MS of the ion at *m*/*z* 693 [M + Na]^+^ as well as at *m*/*z* 97 [HSO_4_]^−^ in the (−)ESIMS/MS of the ion at *m*/*z* 647 [M − Na]^−^ indicated the presence of a sulfate group in **2**. The thorough comparison of the ^1^H and ^13^C NMR data of compound **1** with those of **2** showed that they differed from each other only in position of a sulfate group. The signals H-15 at δ_H_ 3.86 and C-15 at δ_C_ 74.2 in the ^1^H and ^13^C NMR spectra of **2** were shielded when compared with δ_H_ 4.49 and δ_C_ 82.2 in the spectra of **1** that clearly exhibited the lack of a sulfate group at C-15 position. On the other hand, the signals H-4′ at δ_H_ 4.18 and C-4′ at δ_C_ 77.7 of the β-d-xylopyranosyl residue in the ^1^H and ^13^C NMR spectra of **2** were deshielded in comparison to δ_H_ 3.54 and δ_C_ 71.2 in the spectra **1**, respectively, that revealed the presence of a sulfate group at position C-4′ of the β-xylopyranosyl residue in the glycoside **2**. The ^1^H-^1^H COSY, HSQC, HMBC, and NOESY experiments led to the assignment of all proton and carbon resonances in the NMR spectra of **2** (Table 1 and Table 2, Supplementary Materials S8 and S9) and confirmed the conclusion about the 3β,6β,15α-trihydroxysubstituted steroid nucleus and 24-*O*-glycosylated cholestane side chain bearing the 4-*O*-sulfate-β-d-xylopyranosyl unit [19]. The attachment of the monosaccharide to the steroid aglycon was also deduced from long-range correlations in the HMBC and NOESY spectra between H-1′ of 4-*O*-sulfate-β-Xyl_p_ and C-24 or H-24, respectively, of the aglycon. The (*S*)-configuration at C-24 and the d-series of 4-*O*-sulfate-β-xylose unit were expected by analogy with co-occurring glycoside **1**. Thus, the structure of leptaochotensoside B (**2**) was established as (24*S*)-24-*O*-(4-*O*-sulfate-β-d-xylopyranosyl)-5α-cholestane-3β,6β,15α,24-tetraol.

**Table 2:** ^13^C (176.04 MHz) nuclear magnetic resonance (NMR) chemical shifts of **1**–**4** in D_4_-methanol (CD_3_OD), at 30 °C, δ in ppm.

Position	1	2	3	4
1	39.9, CH_2_	38.7, CH_2_	33.6, CH_2_	38.7, CH_2_
2	32.2, CH_2_	32.0, CH_2_	31.7, CH_2_	31.9, CH_2_
3	72.4, CH	72.0, CH	68.3, CH	72.0, CH
4	36.4, CH_2_	33.1, CH_2_	41.5, CH_2_	33.0, CH_2_
5	48.8, CH	52.9, CH	76.6, C	52.8, CH
6	72.6, CH	70.3, CH	76.4, CH	70.3, CH
7	40.2, CH_2_	43.0, CH_2_	35.3, CH_2_	42.9, CH_2_
8	31.3, CH	35.4, CH	31.3, CH	35.4, CH
9	55.6, CH	55.5, CH	46.6, CH	55.4, CH
10	36.6, C	37.4, C	39.3, C	37.3, C
11	22.0, CH_2_	22.3, CH_2_	22.1, CH_2_	22.2, CH_2_
12	41.4, CH_2_	41.6, CH_2_	41.7, CH_2_	41.4, CH_2_
13	44.0, C	44.7, C	44.9, C	44.7, C
14	61.4, CH	63.8, CH	63.5, CH	63.8, CH
15	82.2, CH	74.2, CH	74.3, CH	74.1, CH
16	38.7, CH_2_	41.8, CH_2_	41.8, CH_2_	41.7, CH_2_
17	54.9, CH	55.3, CH	55.1, CH	54.8, CH
18	13.8, CH_3_	14.0, CH_3_	13.8, CH_3_	13.7, CH_3_
19	16.3, CH_3_	14.1, CH_3_	17.4, CH_3_	13.9, CH_3_
20	36.7, CH	36.8, CH	36.8, CH	36.7, CH
21	19.1, CH_3_	18.3, CH_3_	18.3, CH_3_	19.1, CH_3_
22	32.4, CH_2_	33.0, CH_2_	33.0, CH_2_	32.4, CH_2_
23	28.6, CH_2_	28.8, CH_2_	28.6, CH_2_	28.1, CH_2_
24	86.2, CH	86.2, CH	86.1, CH	86.0, CH
25	31.9, CH	32.2, CH	32.0, CH	31.7, CH
26	18.4, CH_3_	18.2, CH_3_	18.2, CH_3_	18.6, CH_3_
27	18.5, CH_3_	19.1, CH_3_	19.1, CH_3_	17.8, CH_3_
1′	105.0, CH	104.6, CH	104.6, CH	
2′	75.3, CH	75.3, CH	75.3, CH	
3′	77.9, CH	76.2, CH	76.1, CH	
4′	71.2, CH	77.8, CH	77.7, CH	
5′	66.7, CH_2_	64.8, CH_2_	64.6, CH_2_	

The molecular formula of **3** was determined to be of С_32_Н_55_О_12_SNa from the [M + Na]^+^ sodiated adduct ion peak at *m*/*z* 709.3182 in the (+)HRESIMS and the [M − Na]^−^ ion peak at *m*/*z* 663.3427 in the (–)HRESIMS. The fragment ion peaks at *m*/*z* 589 [(M + Na) − NaHSO_4_]^+^ in the (+)ESIMS/MS of the ion at *m*/*z* 709 [M + Na]^+^and *m*/*z* 97 [HSO_4_]^−^ in the (−)ESIMS/MS of the ion at *m*/*z* 663 [M − Na]^−^ revealed the presence of a sulfate group in **3**. The molecular mass difference of 16 amu between **3** and **2** assumed the existence of an additional hydroxyl group in **3** compared to glycoside **2**. The comparison of the NMR data of **3** with those of **2** clearly showed that glycoside **3** contained the same 24-*O*-glycosylated cholestane side chain bearing the 4-*O*-sulfate-β-d-xylopyranosyl unit. Most of the signals in the NMR spectra of **3** attributable to steroid nucleus were similar to those of **2** with exception of some resonances belonging to steroid A and B-rings. The signals of H-3α (m) and H_3_-19 (s) in **3** were downfield shifted from δ_H_ 3.47 to 4.00 and from δ_H_ 0.84 to 1.16, respectively, in comparison with those of **2**. The multiplet of H-4β (δ_H_ 2.18) in **2** was replaced by the doublet doublets (δ_H_ 2.05, *J* = 13.0, 11.2 Hz) in the spectrum of **3**. Along with the ^13^C NMR spectrum, these data indicated the presence an additional hydroxyl group at C-5 (δ_C_ 76.6) in **3**. The detailed analysis of the carbon and proton signals and the corresponding coupling constants in the NMR spectra of the aglycon part of **3** testified that compound **3** contained the 3β,5,6β,15α-tetrahydroxysubstituted steroid nucleus (Table 1 and Table 2, Supplementary Materials S10 and S11) [20]. Accordingly, the structure of leptaochotensoside C (**3**) was determined as (24*S*)-24-*O*-(4-*O*-sulfate-β-d-xylopyranosyl)-5α-cholestane-3β,5,6β,15α,24-pentaol.

The molecular formula of compound **4** was determined to be of С_27_Н_47_О_7_SNa from the [M + Na]^+^ sodiated adduct ion peak at *m*/*z* 561.2864 in the (+)HRESIMS and the [M − Na]^−^ ion peak at *m*/*z* 515.3043 in the (−)HRESIMS. The fragment ion peak at *m*/*z* 97 [HSO_4_]^−^ in the (−)ESIMS/MS of the ion at *m*/*z* 515 [M − Na]^−^ indicated the existence of a sulfate group in **4**. Analysis of the ^1^H and ^13^C NMR spectra of **4** and **2** clearly revealed that compound **4** had the same 3β,6β,15α-trihydroxysubstituted steroid nucleus. The protons sequence from H-17 to H-27, correlated with the corresponding carbon atoms of the side chain of **4**, was assigned using the ^1^H-^1^H COSY and HSQC experiments (Table 1 and Table 2, Supplementary Materials S12 and S13). The HMBC correlations H_3_-21/C-17, C-20, C-22; H_3_-26/C-24, C-25, C-27; H_3_-27/C-24, C-25, C-26, and chemical shifts of CH-24 [δ_H_ 4.11 q (*J* = 5.9), δ_C_ 86.0] supported the total structure of the side chain with oxygen-containing function at C-24. Comparison of the carbon and proton resonances and corresponding coupling constants of the side chain with those data reported in literature [20] showed that compound **4** contained the 24-*O*-sulfated cholestane side chain. Thereby, the steroid **4** was proved to be 24-*O*-sulfated aglycon of **2** and its structure was established as (24*S*)-5α-cholestane-3β,6β,15α,24-tetraol 24-*O*-sulfate.

### 2.1. Cytotoxic Activity of Leptaochotensosides A–C (1–3) and Sulfated Polyhydroxysteroid 4 ex Vivo

*Ex vivo* cytotoxicity tests [21] are necessary to define the concentration range for further and more detailed *ex vivo* experiments and to provide meaningful information on signal transduction pathway and molecular targets of compounds.

**Figure 3:**
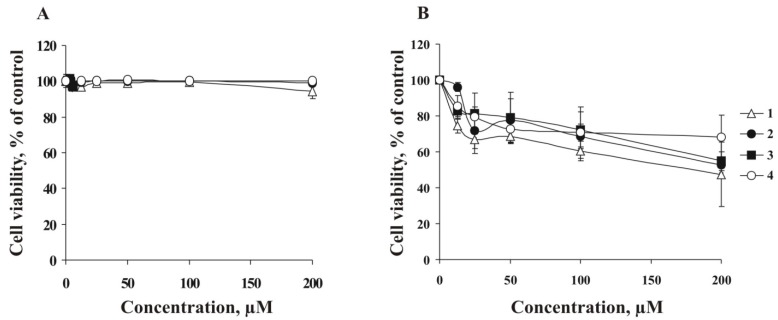
*Ex vivo* cytotoxicities of leptaochotensosides A–C (**1**–**3**) and sulfated polyhydroxysteroid **4**. (**A**) Human melanoma RPMI-7951 cells (8 × 10^3^/well) and (**B**) breast cancer T-47D cells (8 × 10^3^/well) were incubated with compounds **1**–**4** (0–200 µM) for 24 h at 37 °C in an 5% CO_2_ incubator. Compounds cytotoxicities were estimated using MTS assay. Data are represented as the mean ± SD as determined from triplicate experiments.

In the studies on cytotoxic properties of **1**–**4**, human melanoma RPMI-7951 and human breast cancer T-47D cells were treated with various concentrations of **1**–**4** (0–200 µM) for 24 h and then cell viability was assessed by the 3-(4,5-dimethylthiazol-2-yl)-5-(3-carboxymethoxyphenyl)-2-(4-sulfophenyl)-2*H*-tetrazolium (MTS) assay. The results showed that **1**–**4** did not decrease cell viability of RPMI-7951 cells within the concentration range of 0–200 µM (Figure 3A). At the same time, the compounds **1**–**4** possess only slight cytotoxic activity against T-47D cells at the highest of the used concentrations (200 µM) (Figure 3B). For further experiments, RPMI-7951 and T-47D cells were treated with investigated compounds at non-cytotoxic concentrations (lower than 100 μM).

### 2.2. The Effect of Leptaochotensosides A–C (1–3) and Sulfated Polyhydroxysteroid 4 on Colony Formation of Melanoma and Breast Cancer Cells

**Figure 4:**
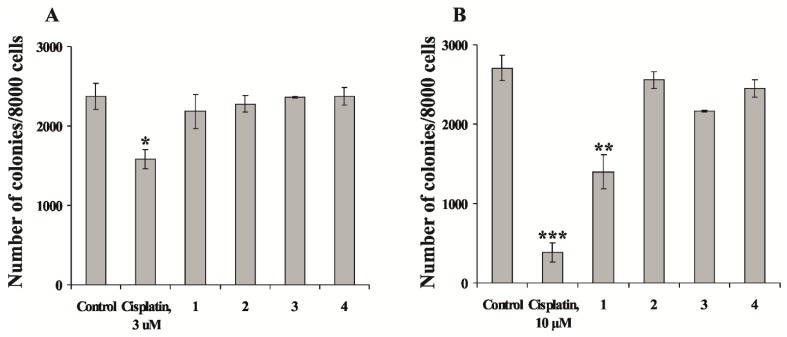
The effect of leptaochotensosides A–C (**1**–**3**) and sulfated polyhydroxysteroid **4** on colony formation of human cancer cells. (**A**) Human melanoma RPMI-7951 cells (2.4 × 10^4^) and (**B**) breast cancer T-47D cells (2.4 × 10^4^) were either treated or not treated with the compounds **1**–**4** in 1 mL of 0.3% Basal Medium Eagle (BME) agar containing 10% fetal bovine serum, 2 mM l-glutamine, and 25 µg/mL gentamicin. The cultures were maintained at 37 °C in an 5% CO_2_ incubator for 14 days and the cell colonies were scored using a microscope Motic AE 20 (Motic, Xiamen, China) and the Motic Image Plus computer program. All assays were performed in at least three independent experiments. Results are expressed as the mean ± standard deviation (SD). Student’s *t*-test was used to evaluate the data with the following significance levels: * *p* < 0.05, ** *p* < 0.01, *** *p* < 0.001.

Next, we determined the effect of compounds **1**–**4** on colony formation of human melanoma RPMI-7951 and human breast cancer T-47D cells using soft agar method. Cells were treated with compounds **1**–**4** at concentration 50 µM and number of colonies was counted after culturing during 14 days. The results showed that compounds **2**–**4** did not inhibit colony formation of RPMI-7951 cells and have slight effect on colony formation of T-47D cells (Figure 4A). The compound **1** also did not effect on colony formation of RPMI-7951 cells (Figure 4A), but significantly reduced colony formation of T-47D cells (the percentage of inhibition of colony formation was 48%) compared with non-treated control (Figure 4B).

Our data indicated that compound **1** at nontoxic doses inhibited the formation of breast cancer cell colonies *ex vivo*.

### 2.3. The Leptaochotensoside A (1) Inhibits the EGF-induced Colony Formation of JB6 Cl41 Cells and Signal Transduction in JB6 Cl41 Cells

The carcinogenesis is multistage process, including initiation (cell transformation), promotion (colony formation), and progression (metastasis) [22]. One of the perspective approaches for cancer therapy is the search and development of nontoxic compounds, which are effective in preventing cancer initiation. The mouse epidermal JB6 Cl41 cells are known to respond irreversibly to tumor promoters such as epidermal growth factor (EGF) by induction of anchorage independent increase in a number of cell colonies in soft agar [23]. That is why this well-established culture system was used to identify the effect of leptaochotensoside A (**1**) on EGF-induced cell transformation.

**Figure 5:**
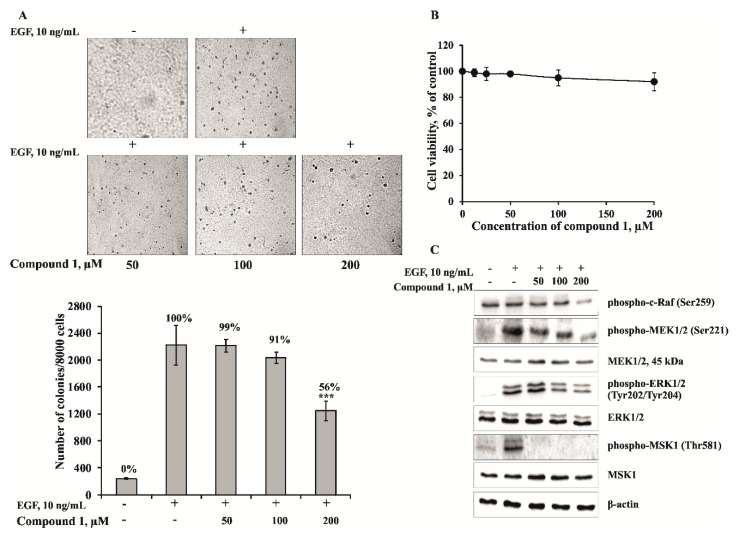
The effect of leptaochotensoside A (**1**) on the epidermal growth factor (EGF)-induced colony formation of JB6 Cl41 cells and molecular mechanism in JB6 Cl41 cells. (**A**) Leptaochotensoside A (**1**) inhibits EGF-induced anchorage-independent growth of mouse epidermal JB6 Cl41 cells. JB6 Cl41 cells (8 × 10^3^) were exposed to EGF (10 ng/mL) and treated with **1** (0–200 µM) in 1 mL of 0.3% Basal Medium Eagle (BME) agar containing 10% fetal bovine serum, 2 mM l-glutamine, and 25 µg/mL gentamicin. The cultures were maintained at 37 °C in an 5% CO_2_ incubator for 14 days, and the cell colonies were scored using a microscope Motic AE 20 (Motic) and the Motic Image Plus computer program. Data are represented as the mean ± SD as determined from triplicate experiments and the asterisks indicate a significant (*** *p* < 0.001) decrease of the colony formation of the cells treated with leptaochotensoside A (**1**) compared with the phosphate buffered saline (PBS)-treated group; (**B**) The absence of cytotoxic effect of leptaochotensoside A (**1**) on JB6 Cl41 cells. An MTS assay was used after treatment of cells with **1** for 24 h. All the experiments were performed in triplicate, and the mean absorbance values were calculated. Data are represented as the mean ± SD as determined from triplicate experiments; (**C**) Leptaochotensoside A (**1**) inhibits MAPK signaling pathway in JB6 Cl41 cells. After cells (6 × 10^5^) were cultured in a 10-cm dish overnight, they were treated with compound **1** (0–200 µM) for 24 h. Then, the cells were starved in serum-free medium for another 12 h and treated with EGF (10 ng/mL) for 15 min. Cells were harvested and protein levels were determined by Western blot analysis.

To estimate the effect of **1**, JB6 Cl41 cells were treated with EGF (10 ng/mL) in the absence or presence of **1** (50, 100, 200 µM) in a soft agar matrix as described in Experimental Section. Our results revealed that JB6 Cl41 cells treated with compound **1** at concentration 200 µM formed less colonies compared with control cells treated with EGF only. It decreased EGF-induced colony number on 44% of control (Figure 5A). The inhibition of by the compound **1** was not due to its cytotoxicity because the effective concentration range for suppressing cell transformation did not affect cell viability of JB6 Cl41 cells (Figure 5B). Since the anchorage-independent growth ability is an *ex vivo* indicator and a key characteristic of the transformed cell phenotype [24], these results suggest that **1** can reduce the malignant potential of JB6 Cl41 cells induced by EGF.

To elucidate cancer prevention molecular mechanism, we tested the effect of leptaochotensoside A (**1**) on activation of mitogen-activated protein kinases (MAPKs). MAPK pathways comprise three kinase modules, in which MAPK is activated upon phosphorylation by a mitogen-activated protein kinase kinase (MAPKK), which in turn is activated, when phosphorylated by MAPKKK [25]. The most thoroughly characterized subgroups of the MAP kinase family include ERKs, c-Jun *N*-terminal kinases (JNKs)/stress-activated protein kinases, and p38 kinases [26]. MAPK pathways are evolutionarily conserved kinase modules that link extracellular signals to the machinery that controls fundamental cellular processes such as growth, proliferation, differentiation, migration and apoptosis [27]. Herein, we describe the effects of compound **1** on the phosphorylation of proto-oncogene serine/threonine-protein (c-Raf), mitogen-activated protein kinase 1/2 (MEK1/2), extracellular signal-regulated 1/2 (ERK1/2), and mitogen- and stress-activated protein (MSK-1) kinases in JB6 Cl41 cells. Compound **1** was shown to inhibit effectively the EGF-induced phosphorylation of ERK1/2 and MSK-1 kinases (Figure 5C). These data suggest that the anticlonogenic effects of compound **1** might be mediated by regulating the activity of MAP kinases, and ERK1/2 and MSK-1 kinases play a key role in its cancer preventive action.

It is well known that steroids such as steroid hormones act through intracellular receptors on gene transcription and protein synthesis (genomic mechanism of action). On the other hand, the regulatory cascades, *i.e.*, MAP kinases, the phosphatidylinositol 3-OH kinase (PI3K) and tyrosine kinases are modulated through non-transcriptional mechanisms by steroid hormones [28,29]. Therefore, we can assume that the action of starfish steroid glycosides on MAPK signal pathway is realized through non-genomic mechanism. The identification of target molecule of leptaochotensoside A (**1**) and elucidation of its binding peculiarity with a target protein or cellular receptor is an aim of further research.

## 3. Experimental Section

### 3.1. General Methods

Optical rotations were determined on a PerkinElmer 343 polarimeter (Perkin Elmer, Waltham, MA, USA). The ^1^H and ^13^C NMR spectra were recorded on Bruker Avance III 700 spectrometers at 700.13 and 176.04 MHz, respectively, with tetramethylsilane used as an internal standard. The HRESI mass spectra were recorded on an Agilent 6510 quadrupole-time of flight liquid chromatography/mass spectrometry (Q-TOF LC/MS) mass spectrometer (Agilent Technologies, Santa Clara, CA, USA); the samples were dissolved in MeOH (c 0.001 mg/mL). GC analysis was performed on an Agilent 6850 Series chromatograph (Agilent Technologies), equipped with a capillary column HP-5 MS (30 m × 0.25 mm) over the temperature range 100–270 °C at 5 °C/min with the carrier gas He (1.7 mL/min); the temperatures of the injector and the detector were 250 and 270 °C, respectively. HPLC separations were carried out on an Agilent 1100 Series chromatograph that was equipped with a differential refractometer; the Diasfer-110-C18 (10 μm, 250 mm × 15 mm), Discovery C_18_ (5 μm, 250 mm × 10 mm), and Diasfer-110-C18 (5 μm, 250 mm × 4.6 mm) columns were used. Low-pressure column liquid chromatography was performed with the Polychrom 1 (powdered Teflon, Biolar, Latvia), silica gel KSK (50–160 μm, Sorbpolimer, Krasnodar, Russia). Sorbfil silica gel plates (4.5 × 6.0 cm, 5–17 μm, Sorbpolimer) were used for thin-layer chromatography.

MTS (3-(4,5-dimethylthiazol-2-yl)-5-(3-carboxymethoxyphenyl)-2-(4-sulfophenyl)-2*H*-tetrazolium) assay kit was purchased from Promega (Madison, WI, USA).

Antibodies against phospho-c-Raf (Ser259), phospho-MEK 1/2 (Ser221), phospho-ERK 1/2 (Tyr202/Tyr204), phospho-MSK 1 (Thr581), MEK 1/2, ERK 1/2, MSK 1 were obtained from Cell Signaling Technology (Danvers, MA, USA), β-actin and horseradish peroxidase (HRP) conjugated secondary antibody from rabbit and mouse were purchased from Santa Cruz Biotechnology (Dallas, TX, USA) and Proteintech Group (Chicago, IL, USA), respectively.

The chemiluminescence’s detection kit ECL Plus was from Amersham (Pittsburgh, PA, USA). The Basal Medium Eagle (BME), Minimum Essential Medium (MEM), Roswell Park Memorial Institute medium (RPMI 1640), phosphate buffered saline (PBS), l-glutamine, gentamicin solution, trypsin, fetal bovine serum (FBS), sodium hydrocarbonate (NaHCO_3_), and agar were purchased from Sigma and Gibco (Carlsbad, CA, USA). All other common chemicals, solvents and reagents were of highest grade available from various commercial sources.

### 3.2. Animal Materials

Specimens of *Leptasterias ochotensis* Brandt, 1851 (order Forcipulatida, family Asteriidae) were collected at a depth of 20–40 m in the Sea of Okhotsk near the Island of Bolshoy Shantar during the research vessel *Akademik Oparin* 29th scientific cruise in August 2003. Species identification was carried out by A.V. Smirnov (Zoological Institute of the Russian Academy of Sciences, St. Petersburg, Russia). A voucher specimen (no. 029-052) is on deposit at the marine specimen collection of the G.B. Elyakov Pacific Institute of Bioorganic Chemistry of the FEB RAS, Vladivostok, Russia.

### 3.3. Extraction and Isolation

The fresh animals (0.35 kg) were chopped and extracted twice with EtOH at 20 °C. The H_2_O/EtOH layer was evaporated, and the residue was dissolved in H_2_O (0.5 L). The H_2_O-soluble materials were passed through a Polychrom 1 column (6.5 cm × 21 cm), eluted with distilled H_2_O until a negative chloride ion reaction was obtained, and then eluted with EtOH. The combined EtOH eluate was evaporated to give a brownish residue (4.8 g). This material was chromatographed over a silica gel column (4 cm × 20 cm) using CHCl_3_/EtOH (stepwise gradient, 8:1 to 1:2) to yield six fractions, 1–6, that were then analyzed by TLC on silica gel plates in the eluent system BuOH/EtOH/H_2_O (4:1:2). Fractions 1, 2, 4, and 5 mainly contained the sulfated polyhydroxysteroids and related glycosides, fraction 3 mainly contained the free sulfated asterogenins, and fraction 6 mainly contained the asterosaponins. HPLC separation of fraction 5 on a Diasfer-110-C18 column (10 μm, 250 mm × 15 mm, 2.5 mL/min) with EtOH/H_2_O (65:35) as an eluent system followed by the further separation on a Discovery C_18_ column (5 μm, 250 mm × 10 mm, 2.5 mL/min) with MeOH/H_2_O (70:30) as an eluent system yielded pure **1** (38.6 mg, *t*_R_ 18.7 min) and several additional subfractions, containing compounds **2**–**4**. Further HPLC separation of these subfractions on a Diasfer-110-C18 column (5 μm, 250 mm × 4.6 mm, 1.0 mL/min) with MeOH/H_2_O (75:25) as an eluent system yielded pure **2** (1.5 mg, *t*_R_ 7.4 min), **3** (7.5 mg, *t*_R_ 6.8 min), and **4** (1.7 mg, *t*_R_ 11.2 min).

### 3.4. Spectral Data of New Compounds

Leptaochotensoside A (**1**): С_32_Н_55_О_11_SNa, amorphous powder; ${\left[\alpha \right]}_{D}^{25}$ +12.3 (*с* 0.45, MeOH); the ^1^H and ^13^C NMR data are listed in Table 1 and Table 2; (+)HRESIMS *m*/*z* 693.3225 [M + Na]^+^ (calculated for C_32_H_55_O_11_SNa_2_, 693.3255). (+)ESIMS/MS of the ion [M + Na]^+^ at *m*/*z* 693: 573 [(M + Na) − NaHSO_4_]^+^, 543 [(M + Na) − С_5_H_8_O_4_ − H_2_O]^+^, 143 [Na_2_HSO_4_]^+^. (−)HRESIMS *m*/*z* 647.3485 [M − Na]^−^ (calculated for C_32_H_55_O_11_S, 647.3471). (–)ESIMS/MS of the ion [M − Na]^−^ at *m*/*z* 647: 629 [(M − Na) − H_2_O]^−^, 515 [(M − Na) − С_5_H_8_O_4_]^−^, 497 [(M − Na) − С_5_H_8_O_4_ − H_2_O]^−^, 97 [HSO_4_]^−^.

Leptaochotensoside B (**2**): C_32_H_55_O_11_SNa, amorphous powder; ${\left[\alpha \right]}_{D}^{25}$ +3.5 (*с* 0.1, MeOH); the ^1^H and ^13^C NMR data are listed in Table 1 and Table 2; (+)HRESIMS *m*/*z* 693.3238 [M + Na]^+^ (calculated for C_32_H_55_O_11_SNa_2_, 693.3255). (+)ESIMS/MS of the ion [M + Na]^+^ at *m/z* 693: 573 [(M + Na) − NaHSO_4_]^+^. (−)HRESIMS *m*/*z* 647.3480 [M − Na]^−^ (calculated for C_32_H_55_O_11_S, 647.3471). (−)ESIMS/MS of the ion [M − Na] ^−^ at *m*/*z* 647: 97 [HSO_4_] ^−^.

Leptaochotensoside C (**3**): C_32_H_55_O_12_SNa, amorphous powder; ${\left[\alpha \right]}_{D}^{25}$ +3.5 (*с* 0.1, MeOH); the ^1^H and ^13^C NMR data are listed in Table 1 and Table 2; (+)HRESIMS *m*/*z* 709.3182 [M + Na]^+^ (calculated for C_32_H_55_O_12_SNa_2_, 709.3204). (+)ESIMS/MS of the ion [M + Na]^+^ at *m*/*z* 709: 589 [(M + Na) − NaHSO_4_]^+^. (−)HRESIMS *m*/*z* 663.3427 [M − Na]^−^ (calculated for C_32_H_55_O_12_S, 663.3420). (−)ESIMS/MS of the ion [M − Na]^−^ at *m*/*z* 663: 97 [HSO_4_]^−^.

(24*S*)-5α-Cholestane-3β,6β,15α,24-tetraol 24-*O*-sulfate (**4**): C_27_H_47_O_7_SNa, amorphous powder; ${\left[\alpha \right]}_{D}^{25}$ +7.0 (*с* 0.1, MeOH); the ^1^H and ^13^C NMR data are listed in Table 1 and Table 2; (+)HRESIMS *m*/*z* 561.2864 [M + Na]^+^ (calculated for C_27_H_47_O_7_SNa_2_, 561.2838). (−)HRESIMS *m*/*z* 515.3043 [M − Na]^−^ (calculated for C_27_H_47_O_7_S, 515.3042). (−)ESIMS/MS of the ion [M − Na]^−^ at *m*/*z* 515: 97 [HSO_4_]^−^.

### 3.5. Acid Hydrolysis and Determination of Absolute Configuration of Monosaccharide

The acid hydrolysis of **1** (2.0 mg) was carried out in a solution of 2 M TFA (1 mL) in a sealed vial on a H_2_O bath at 100 °C for 2 h. The H_2_O layer was washed with CHCl_3_ (3 × 1.0 mL) and concentrated *in vacuo*. One drop of concentrated TFA and 0.5 mL of *R*-(−)-2-octanol (Aldrich) were added to the sugar mixture, and the sealed vial was heated on a glycerol bath at 130 °C for 6 h. The solution was evaporated *in vacuo* and treated with a mixture of pyridine/acetic anhydride (1:1, 0.6 mL) for 24 h at room temperature. The acetylated 2-octylglycosides were analyzed by GC using the corresponding authentic samples prepared by the same procedure. The following peaks of monosaccharide unit were detected in the hydrolysate of **1**: d-xylose (*t*_R_ 24.32, 24.55, and 24.80 min). The retention times of the authentic samples were as follows: d-xylose (*t*_R_ 24.28, 24.57, and 24.78 min) and l-xylose (*t*_R_ 24.07, 24.15, 24.71, and 24.92 min).

### 3.6. Bioactivity Assay

#### 3.6.1. Cell Lines and Culture Conditions

Mouse epidermal JB6 Cl41 cells (АТСС # CRL-2010™), human malignant melanoma RPMI-7951 cells (ATCC # HTB-66™), and human breast cancer cells T-47D (ATCC # HTB-133™) were obtained from the American Type Culture Collection (ATCC, Manassas, VA, USA).

The JB6 Cl41, RPMI-7951, and T-47D cells were cultured in MEM/5% FBS, MEM/10% FBS, and RPMI-1640/10% FBS media, respectively. The cell cultures were maintained at 37 °C in an 5% CO_2_ incubator (MCO-18AIC, Sanyo, Moriguchi, Osaka, Japan). The cells were grown for 3–4 days and after reaching 90% of confluence were harvested by exposure to 0.25% trypsin-ethylenediaminetetraacetic acid (trypsin-EDTA) solution and then passed into new T-75 tissue culture flasks.

#### 3.6.2. MTS Assay

To estimate cell viability, JB6 Cl41, RPMI-7951, and T-47D cells (8 × 10^3^/well) were seeded in 96-well plates for 24 h at 37 °C in 5% CO_2_ incubator. The attached cells were fed with fresh medium containing various concentrations of compounds **1**–**4** from *L. ochotensis* (0–200 µM) for additional 24 h. After that, the cytotoxicity of **1**–**4** was measured using an MTS assay kit according to the manufacturer’s instructions. All the experiments were performed in triplicate, and the mean absorbance values were calculated. The results are expressed as the percentage of inhibition that produced a reduction in absorbance after treatment with polar steroids compared to the non-treated cells (control).

#### 3.6.3. Anchorage-Independent Cell Transformation Assay (Soft Agar)

Human melanoma RPMI-7951 (2.4 × 10^4^) and breast cancer T-47D (2.4 × 10^4^) cells were treated with compounds **1**–**4** at nontoxic dose (50 µM) in 1 mL of 0.3% BME agar containing 10% FBS, 2 mM l-glutamine, and 25 µg/mL gentamicin. The cultures were maintained at 37 °C in an 5% CO_2_ incubator for 14 days, and the cell colonies were scored using a microscope Motic AE 20 (Motic) and the Motic Image Plus computer program.

To estimate the effect of compound **1** on the EGF-induced growth of the cell colonies, mouse epidermal JB6 Cl41 cells (2.4 × 10^4^) were exposed to EGF (10 ng/mL) and treated with **1** (50, 100, 200 µM) in 1 mL of 0.3% BME agar containing 10% FBS, 2 mM l-glutamine, and 25 µg/mL gentamicin. The cultures were maintained at 37 °C in a 5% CO_2_ incubator for 14 days, and the cell colonies were scored as described above.

#### 3.6.4. Western Blot Analysis

After JB6 Cl41 cells (6 × 10^5^) were cultured in a 10-cm dish overnight, they were treated with **1** (50, 100, 200 µM) for 24 h. Then, the cells were starved in serum-free medium for another 12 h and treated with EGF (10 ng/mL) for 15 min. The harvested cells were lysed with lysis buffer (50 mM Tris-HCl (pH 7.4), 150 mM·NaCl, 1 mM EDTA, 1 mM ethylene glycol tetraacetic acid (EGTA), 10 mg/mL aprotinin, 10 mg/mL leupeptin, 5 mM phenylmethanesulfonyl fluoride (PMSF), 1 mM dithiolthreitol (DTT) containing 1% Triton X-100). Insoluble debris was removed by centrifugation at 12,000 rpm for 15 min, and protein’s content was determined using Bradford reagent (Bio-Rad, Hercules, CA, USA). Lysate protein (20–40 µg) was subjected to 10% sodium dodecyl sulfate-polyacrylamide gel electrophoresis (SDS-PAGE) and electrophoretically transferred to a polyvinylidene difluoride membranes (PVDF) (Millipore, Billerica, MA, USA). The membranes were blocked with 5% non-fat milk for 1 h and then incubated with the respective specific primary antibody at 4 °C overnight. Protein bands were visualized using an enhanced chemiluminescence reagent (ECL Plus, GE Healthcare, Marlborough, MA, USA) after hybridization with a HRP-conjugated secondary antibody. Band density was quantified using the ImageJ software program (National Institutes of Health, Bethesda, MD, USA).

#### 3.6.5. Statistical Analysis

All assays were performed at least in triplicate. The results are expressed as the mean ± standard deviation (SD). A Student’s *t*-test was used to evaluate the data with the following significance levels: * *p* < 0.05, ** *p* < 0.01, *** *p* < 0.001.

## 4. Conclusions

Four new sulfated polyhydroxysteroidal compounds (**1**–**4**) were isolated from the alcoholic extract of the Far Eastern starfish *Leptasterias ochotensis* and their chemical structures were elucidated by extensive NMR and ESIMS techniques. The biological action of new leptaochotensosides A–C (**1**–**3**) and sulfated steroid (**4**) were examined using the EGF-induced colony formation of normal mouse epidermal cells and the growth and colony formation of human melanoma and breast cancer cells. Our results indicated that compounds **2**–**4** at non-toxic doses did not inhibit colony formation of RPMI-7951 cells and have slight cytotoxic effect on colony formation of T-47D cells. Leptaochotensoside A (**1**) was able to inhibit colony formation of T-47D cells and the EGF-induced colony formation of JB6 Cl41 cells. Compound **1** demonstrated its antiproliferative effects in part through the inhibition of phosphorylation of MAP kinases, namely through the inhibition of EGF-induced phosphorylation of ERK1/2 and MSK-1 kinases.

## Supplementary Files

Supplementary File 1
