# Supplementary material for: Four New Sulfated Polar Steroids from the Far Eastern Starfish Leptasterias ochotensis: Structures and Activities
Source: Mar Drugs. 2015 Jul 16;13(7):4418–35. doi: 10.3390/md13074418 (PMC4515625; doi:10.3390/md13074418)
Supplement: Supplementary File 1 [file marinedrugs-13-04418-s001.doc]

# Supplementary Information


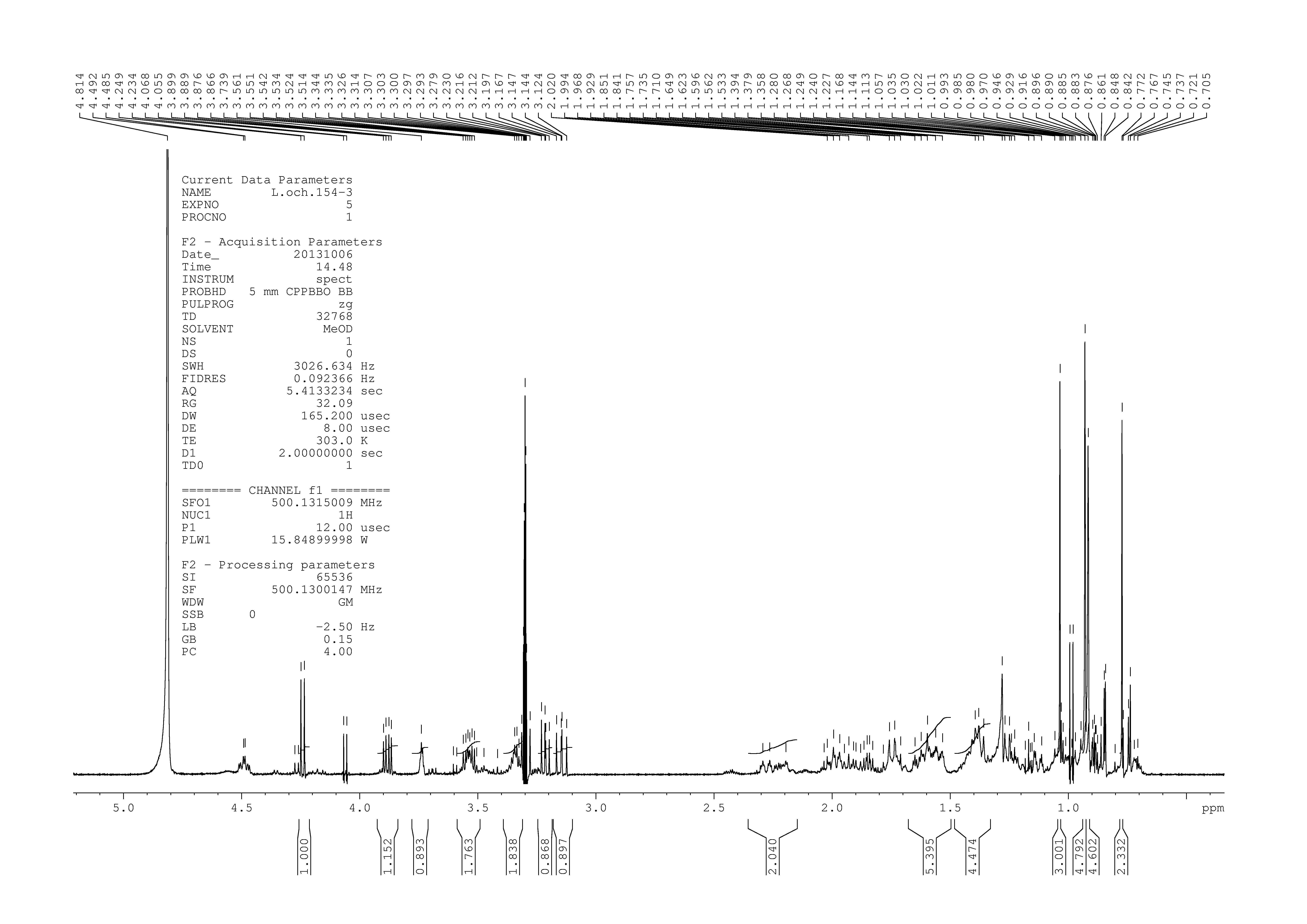


**Figure S1.** ^1^H NMR (nuclear magnetic resonance) spectrum of compound **1** in D_4_-methanol (CD_3_OD).


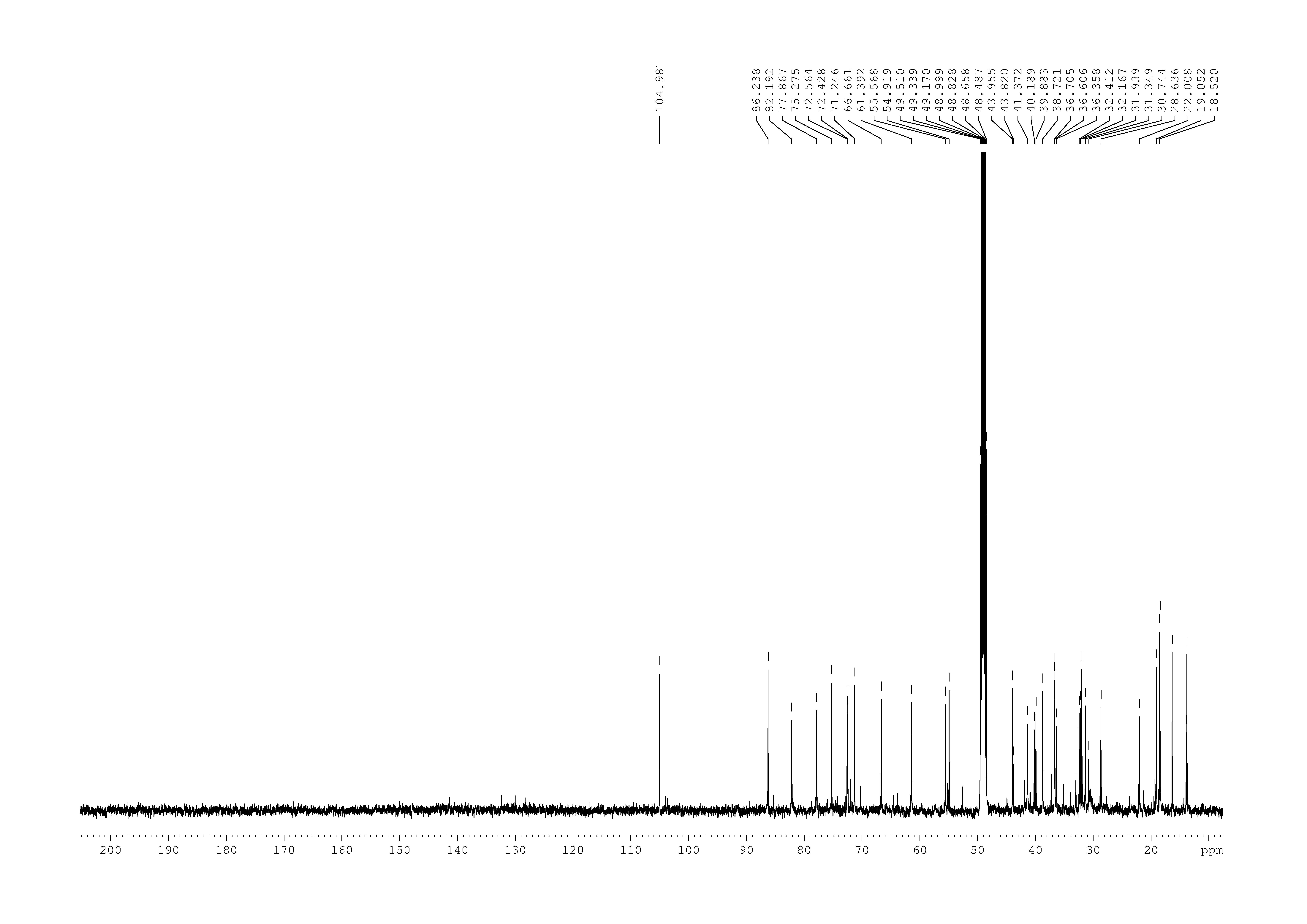


**Figure S2.** ^13^C NMR (nuclear magnetic resonance) spectrum of compound **1** in D_4_-methanol (CD_3_OD).


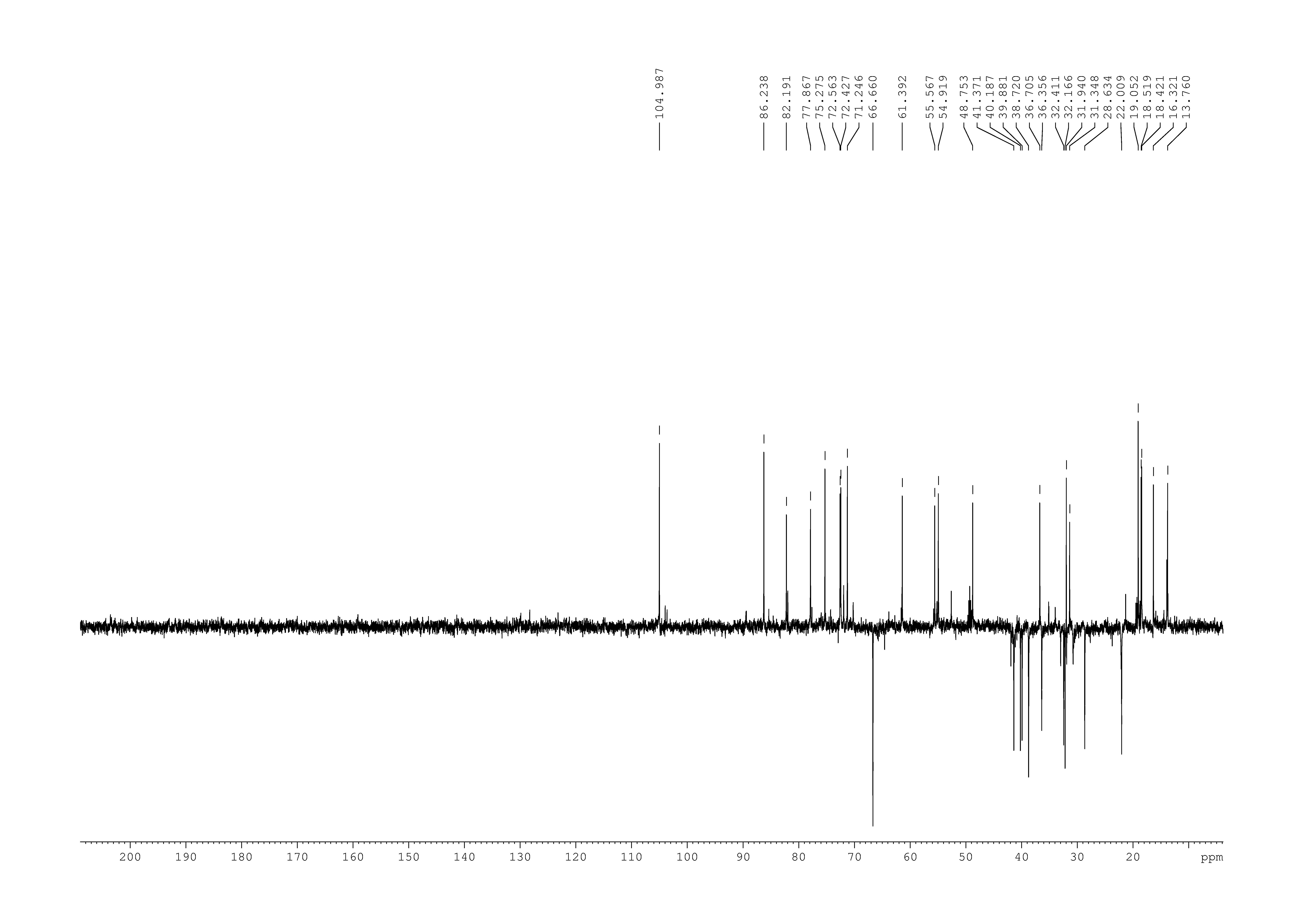


**Figure S3.** Distortionless enhancement by polarization transfer (DEPT) spectrum of compound **1** in D_4_-methanol (CD_3_OD).


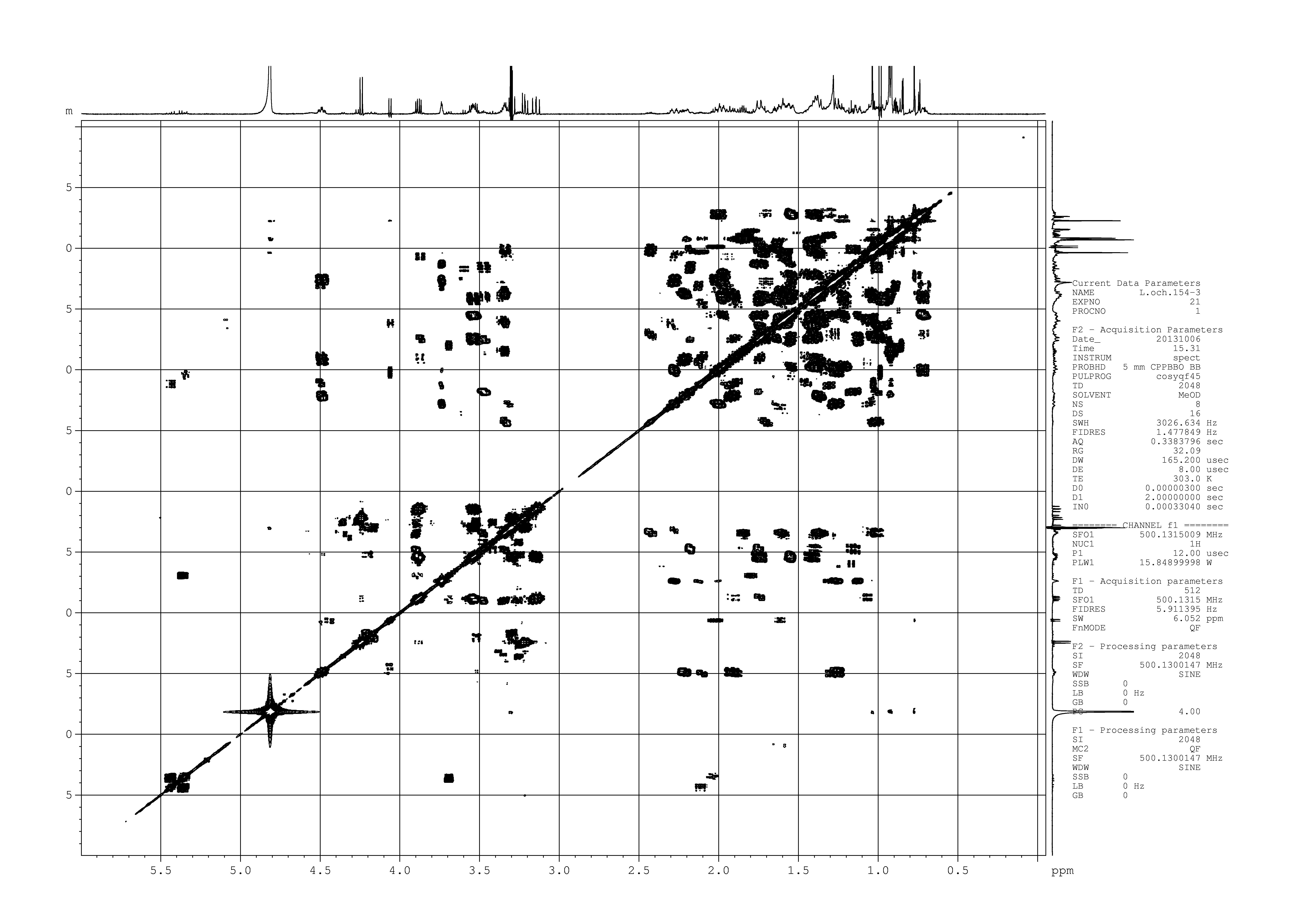


**Figure S4.** ^1^H-^1^H Correlation spectroscopy (^1^H-^1^H COSY) spectrum of compound **1** in D_4_-methanol (CD_3_OD).


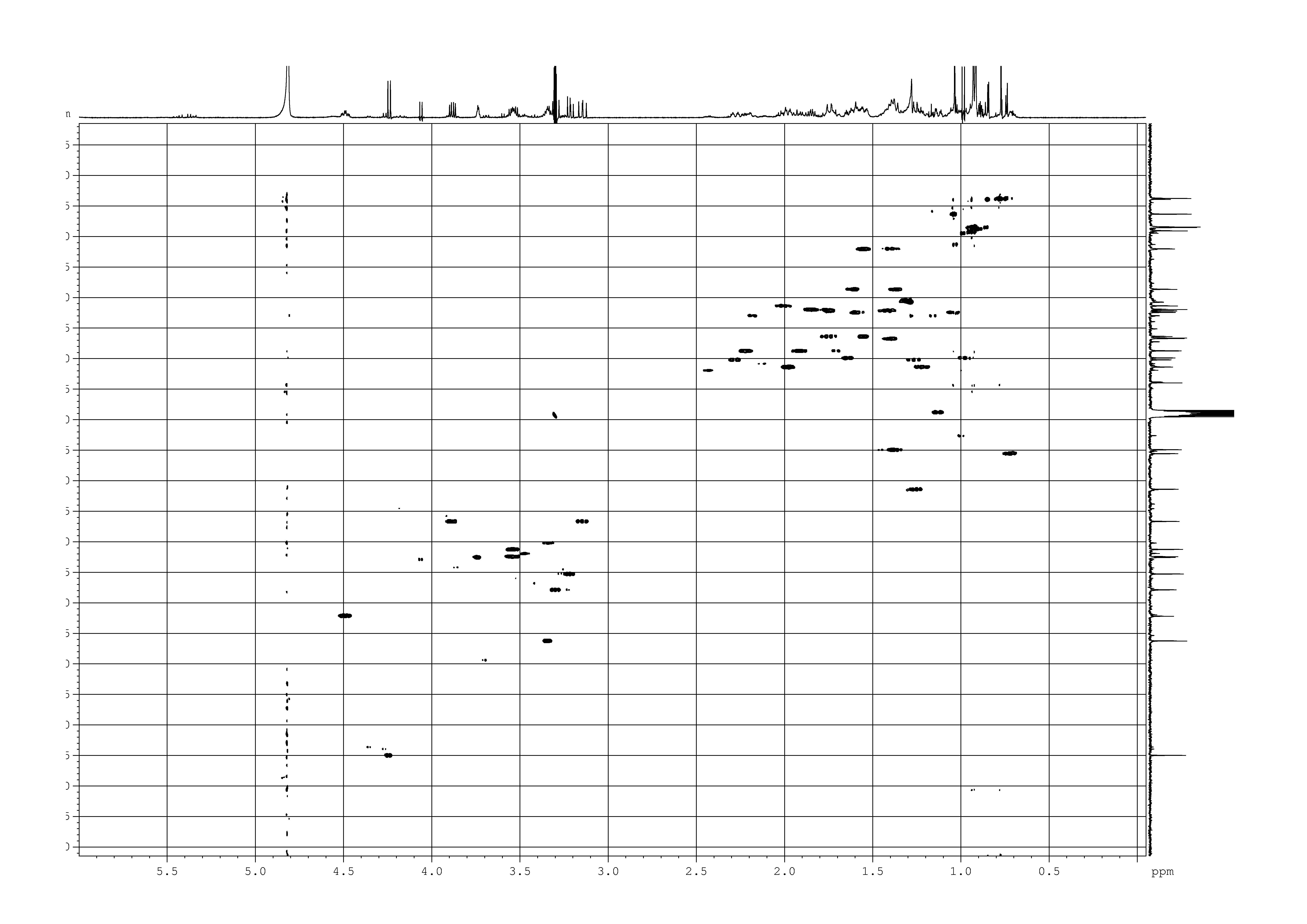


**Figure S5.** Heteronuclear single quantum connectivity (HSQC) spectrum of compound **1** in D_4_-methanol (CD_3_OD).


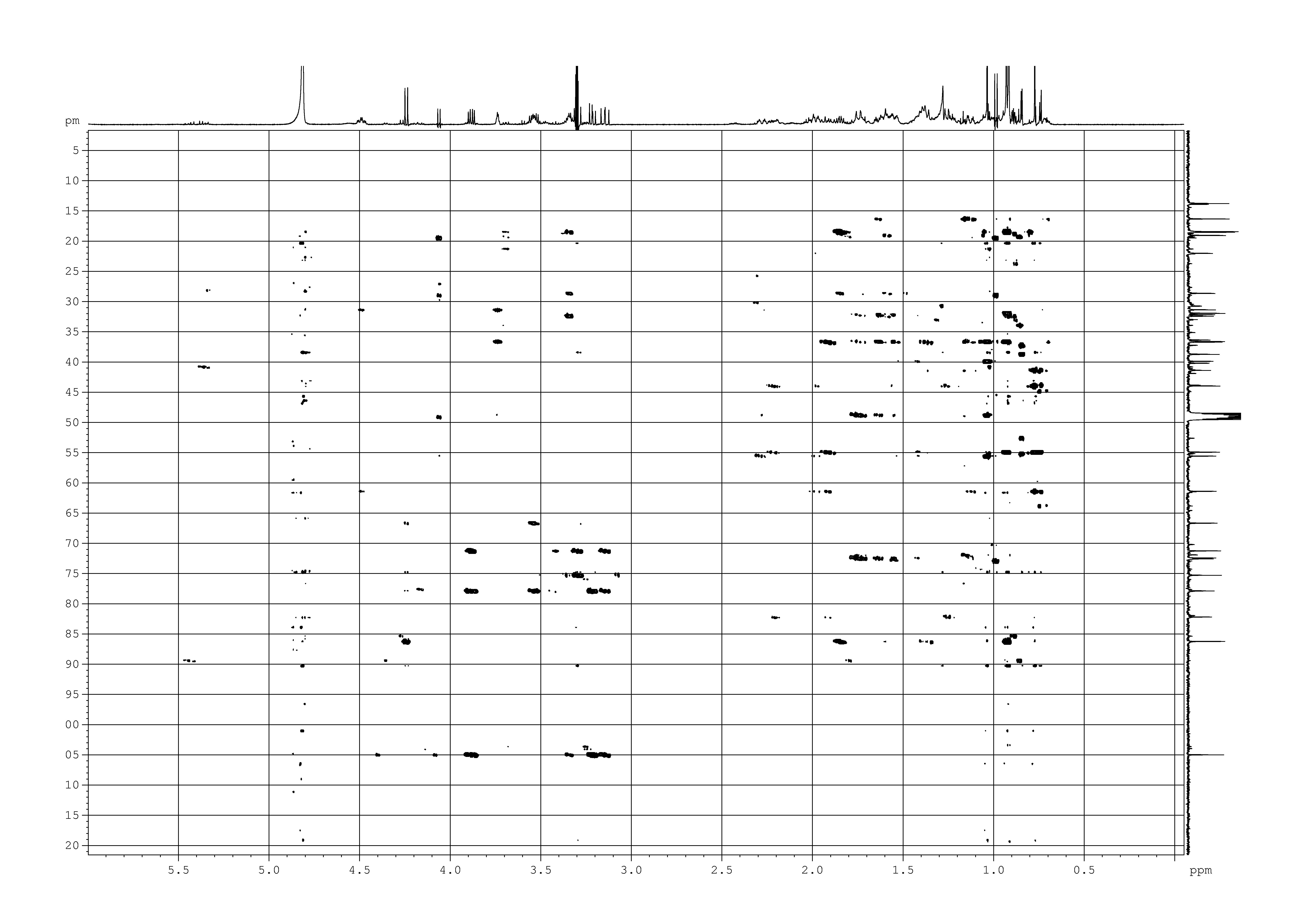


**Figure S6.** Heteronuclear multiple bond connectivity (HMBC) spectrum of compound **1** in D_4_-methanol (CD_3_OD).


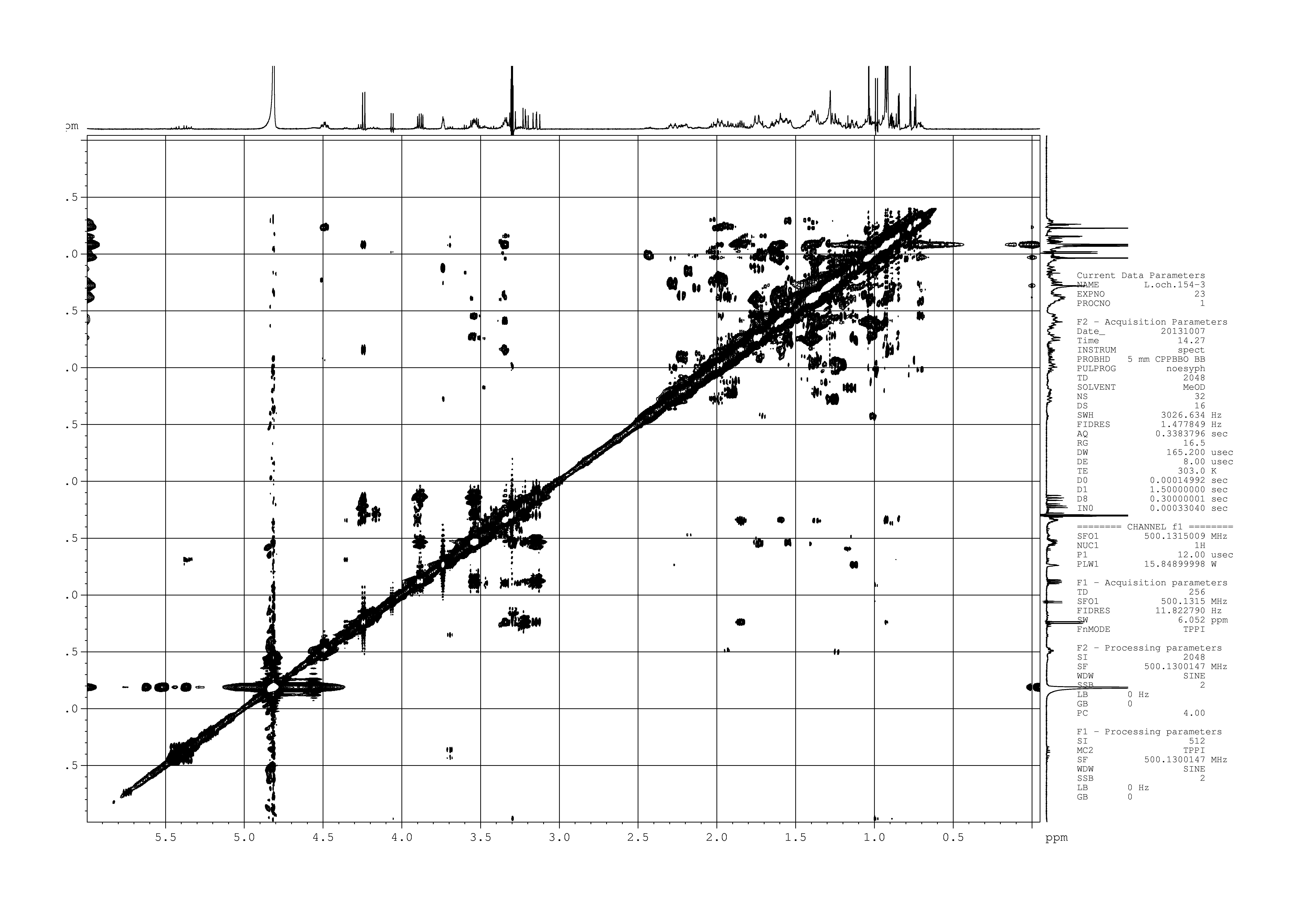


**Figure S7.** Nuclear Overhauser effect spectroscopy (NOESY) spectrum of compound **1** in D_4_-methanol (CD_3_OD).


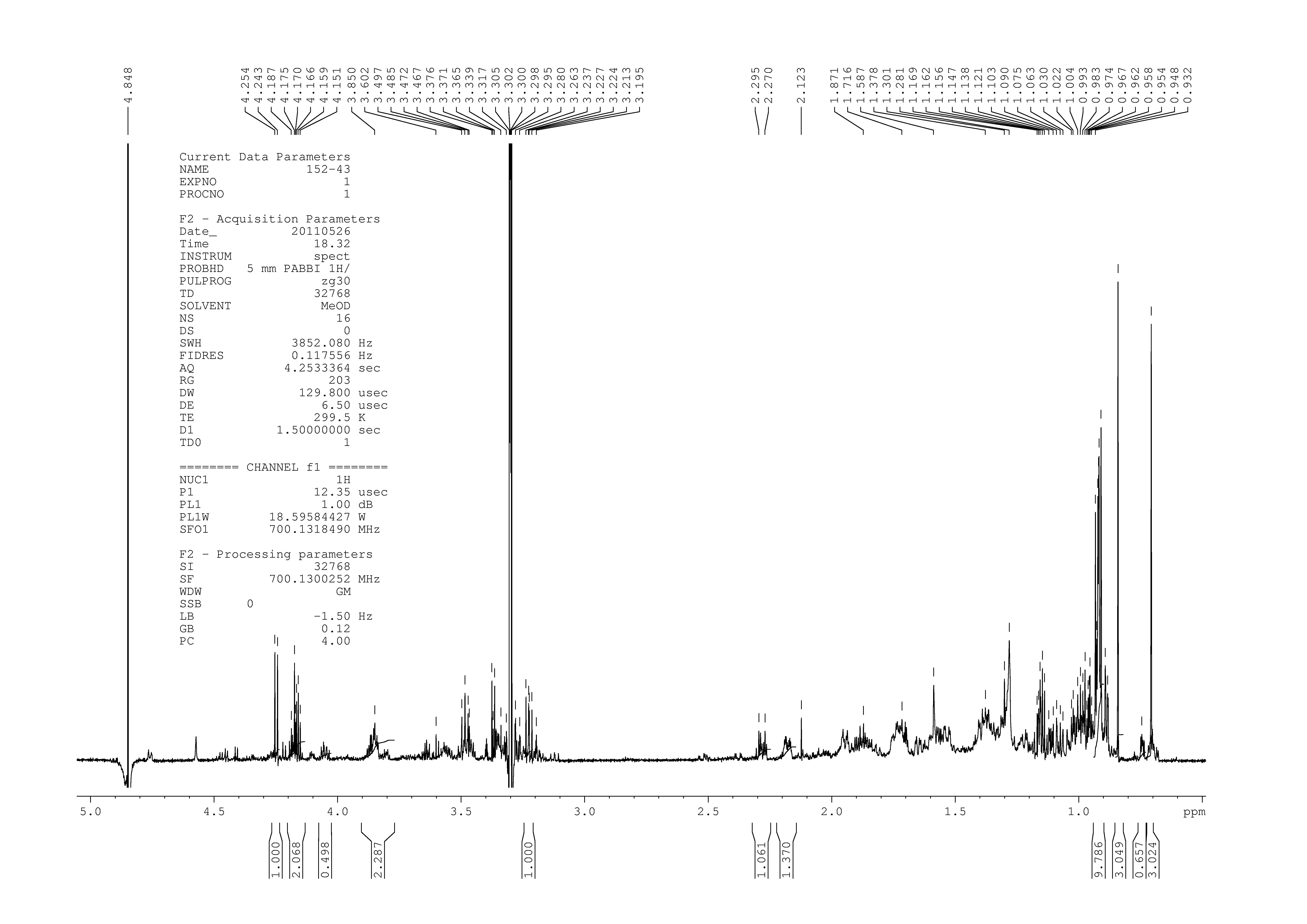


**Figure S8.** ^1^H NMR (nuclear magnetic resonance) spectrum of compound **2** in D_4_-methanol (CD_3_OD).


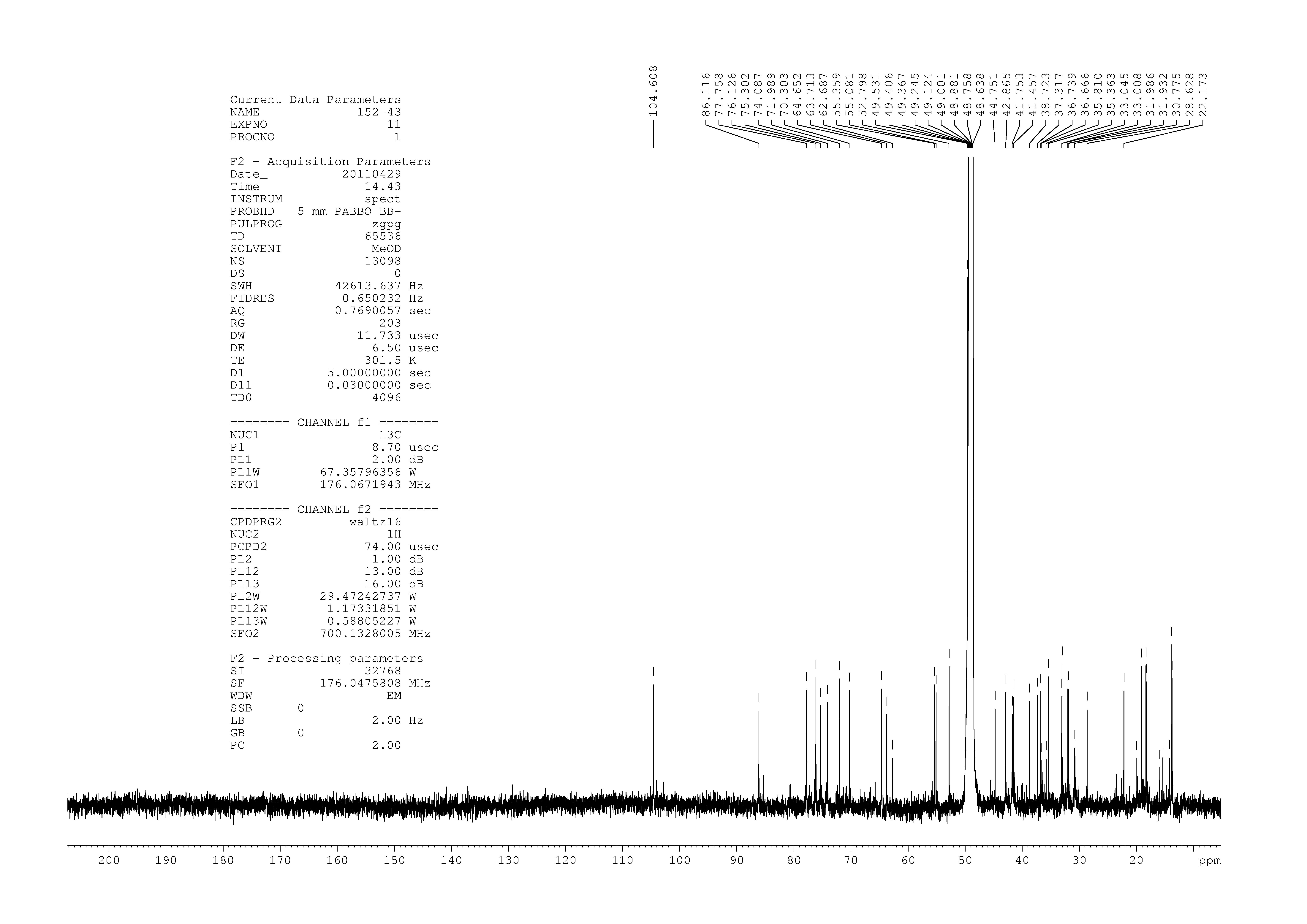


**Figure S9.** ^13^C NMR (nuclear magnetic resonance) spectrum of compound **2** in D_4_-methanol (CD_3_OD).


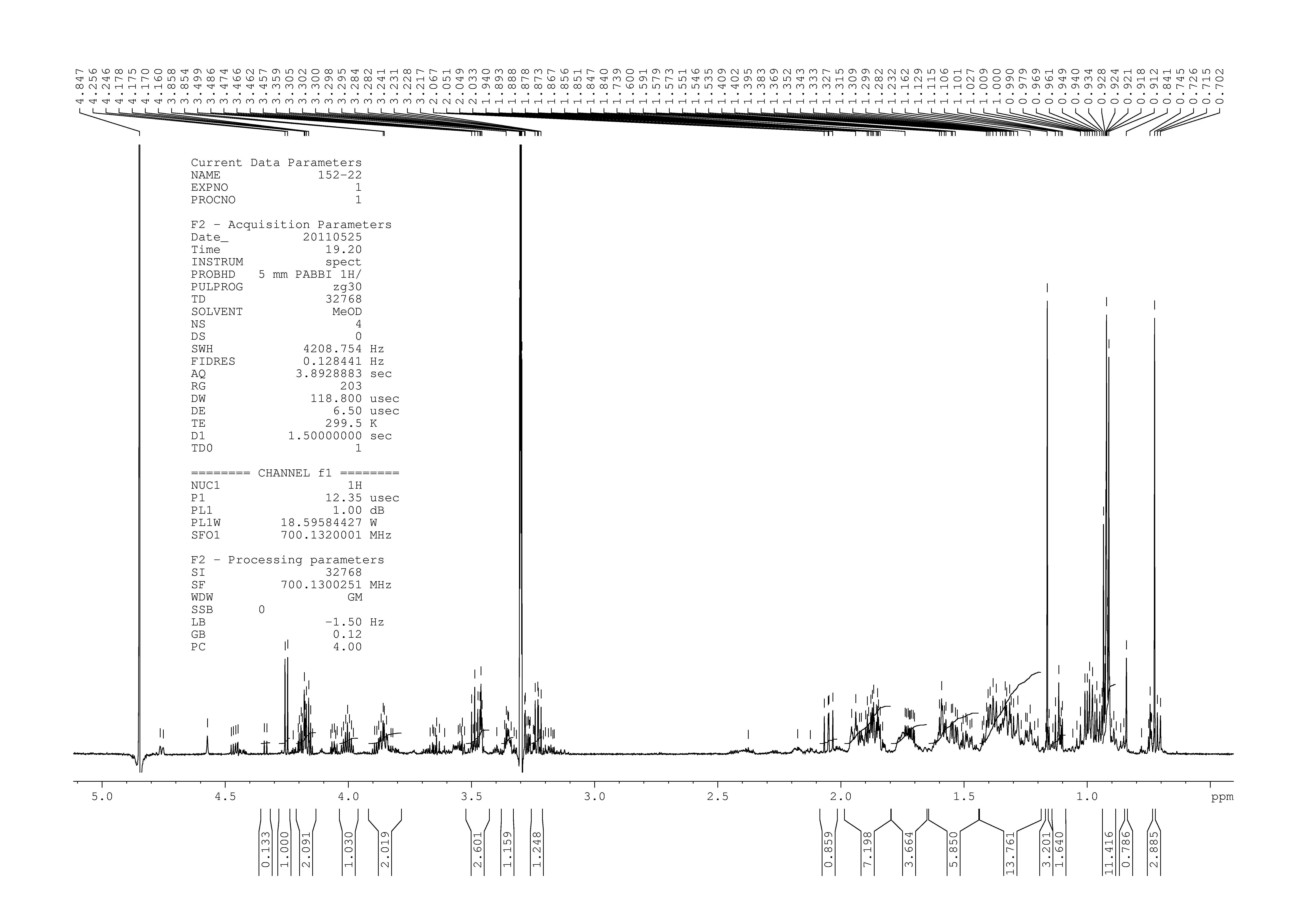


**Figure S10.** ^1^H NMR (nuclear magnetic resonance) spectrum of compound **3** in D_4_-methanol (CD_3_OD).


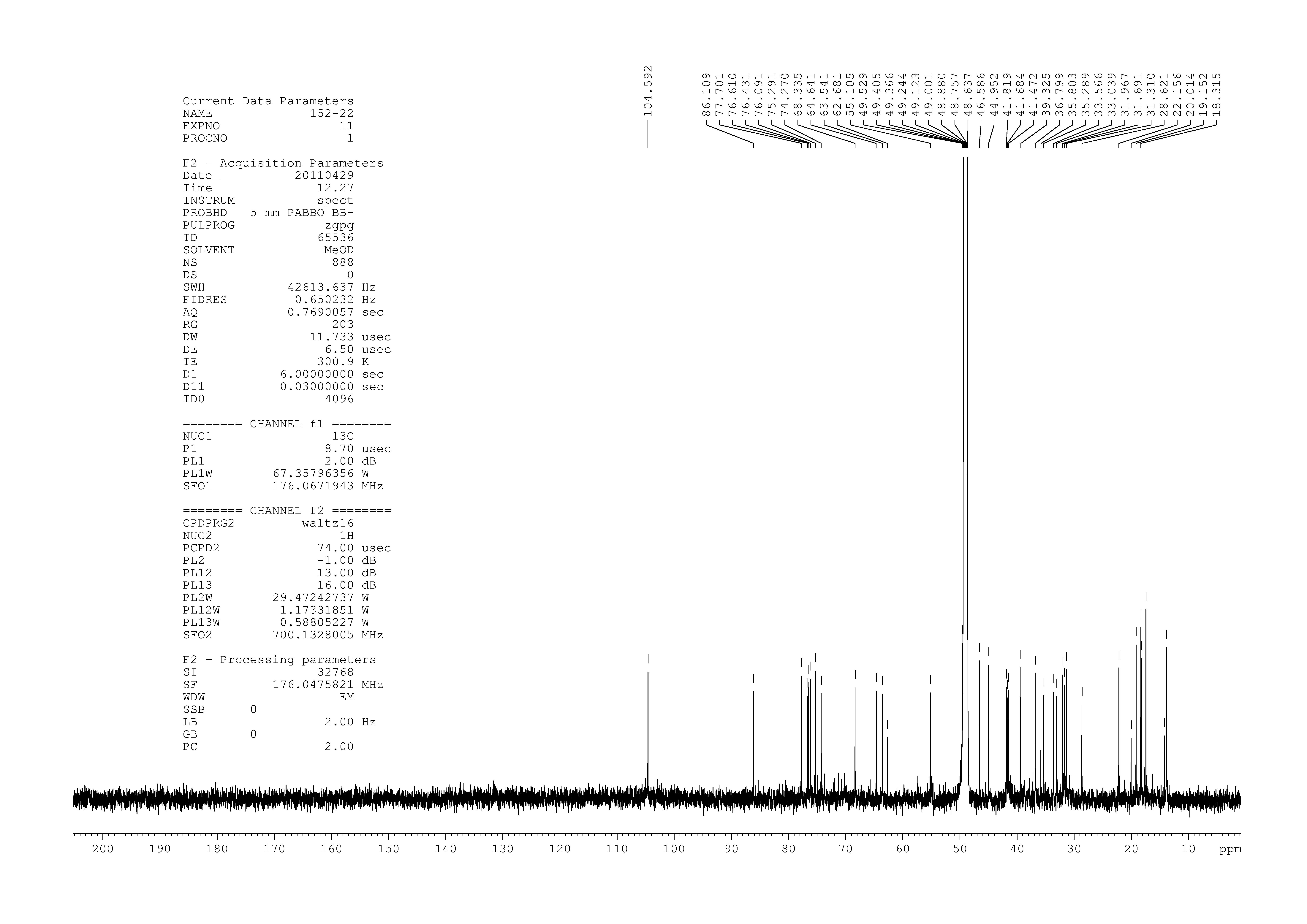


**Figure S11.** ^13^C NMR (nuclear magnetic resonance) spectrum of compound **3** in D_4_-methanol (CD_3_OD).


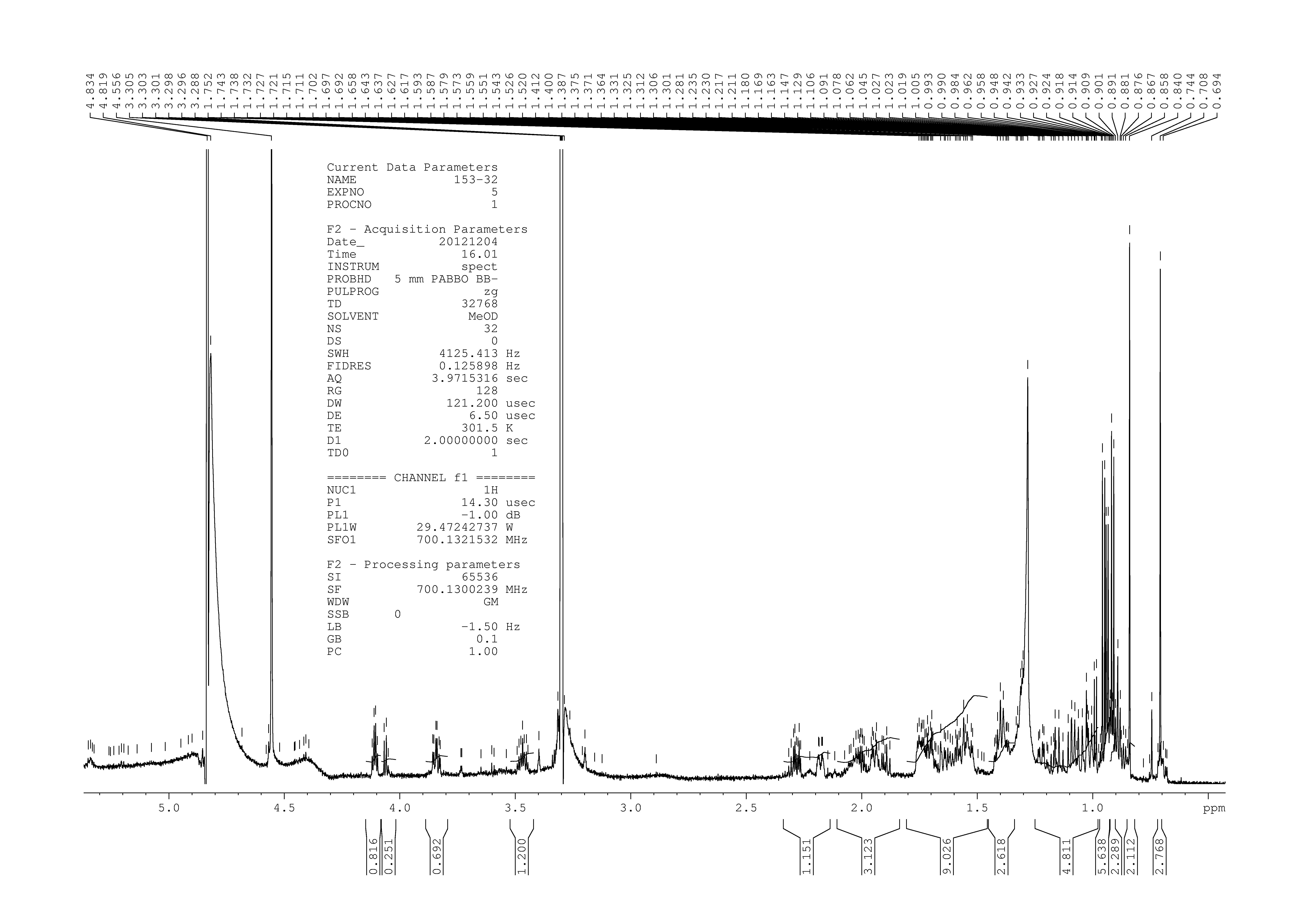


**Figure S12.** ^1^H NMR (nuclear magnetic resonance) spectrum of compound **4** in D_4_-methanol (CD_3_OD).


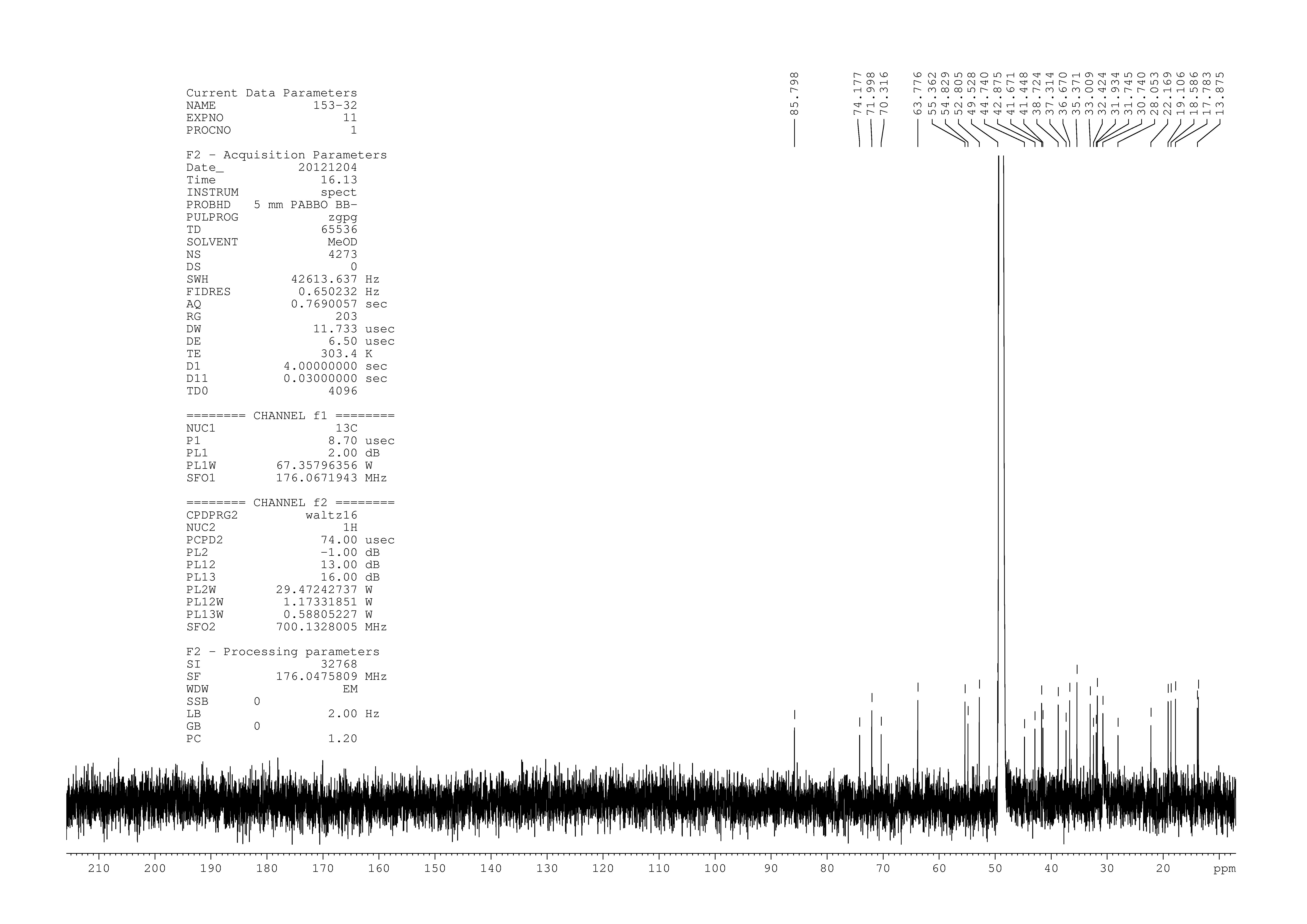


**Figure S13.** ^13^C NMR (nuclear magnetic resonance) spectrum of compound **4** in D_4_-methanol (CD_3_OD).

© 2015 by the authors; licensee MDPI, Basel, Switzerland. This article is an open access article distributed under the terms and conditions of the Creative Commons Attribution license (http://creativecommons.org/licenses/by/4.0/).
